# Nanoparticles to Improve the Efficacy of Peptide-Based Cancer Vaccines

**DOI:** 10.3390/cancers12041049

**Published:** 2020-04-23

**Authors:** Anna Lucia Tornesello, Maria Tagliamonte, Maria Lina Tornesello, Franco M. Buonaguro, Luigi Buonaguro

**Affiliations:** 1 Molecular Biology and Viral Oncology Unit, Istituto Nazionale Tumori IRCCS “Fondazione G. Pascale”, via Mariano Semmola, 80131 Napoli, Italy; m.tornesello@istitutotumori.na.it (M.L.T.); f.buonaguro@istitutotumori.na.it (F.M.B.); 2 Innovative Immunological Models, Istituto Nazionale Tumori IRCCS “Fondazione G. Pascale”, via Mariano Semmola, 80131 Napoli, Italy; m.tagliamonte@istitutotumori.na.it

**Keywords:** cancer vaccines, peptide-based vaccine, nanoparticles, tumor vaccines, CPPs, VLPs

## Abstract

Nanoparticles represent a potent antigen presentation and delivery system to elicit an optimal immune response by effector cells targeting tumor-associated antigens expressed by cancer cells. Many types of nanoparticles have been developed, such as polymeric complexes, liposomes, micelles and protein-based structures such as virus like particles. All of them show promising results for immunotherapy approaches. In particular, the immunogenicity of peptide-based cancer vaccines can be significantly potentiated by nanoparticles. Indeed, nanoparticles are able to enhance the targeting of antigen-presenting cells (APCs) and trigger cytokine production for optimal T cell response. The present review summarizes the categories of nanoparticles and peptide cancer vaccines which are currently under pre-clinical evaluation.

## 1. Introduction

Cancer vaccines are based on tumor antigens administered as nucleic acids, tumor lysates, full proteins or short peptides. In particular, peptides are protein subunits presented by antigen-presenting cells (APCs) to elicit cell-mediated immunity. Indeed, APCs, such as dendritic cells (DCs), are able to capture, internalize and process into short peptides, proteins expressed by viruses or tumor cells. 

Peptides are then loaded to MHC class I and MHC class II molecules. The peptide-MHC-I complex is recognized by CD8^+^ T cells which are activated to exert their cytotoxic activity on tumor cells presenting the same peptide-MHC-I complex. 

In parallel, the peptide-MHC-II complex interacts with CD4^+^ T helper cells which may differentiate in two major subtype, Th2 and Th1, that are involved in inflammatory response and in potentiating and sustaining the activity of CD8^+^ T (CTLs), respectively [1]. Therefore, the development of a cancer vaccine requires an optimal targeting of DCs for an efficient antigen presentation to CD4^+^ T helper cells. This can be achieved by either modifying the antigen structure, or conjugation with specific peptide sequences (cell penetrating peptides, CPPs) or loading nanoparticles. Moreover, the use of adjuvants allows to potentiate the antigen immunogenity and to drive the differentiation of Th cells towards a Th1 pattern [2].

Peptides used for vaccine formulations are 15–30 amino acid-long sequences corresponding to the relevant epitopes for antigenic recognition by T cells. Therefore, they represent an alternative strategy compared to standard vaccine formulations which are mainly based on attenuated or killed whole pathogens, toxoids or carbohydrates. Peptide-based vaccines have a low risk of pathogenic effects or off target responses and are very versatile being formulated as synthetic peptides or encoded by DNA or RNA molecules [3].

Peptide-based cancer vaccines aim at expanding pre-existing immunity as well as at inducing de novo antitumor T cell responses in cancer patients [4]. High throughput technologies for synthetic peptide production allows the introduction of several modifications in the peptide linear sequence to refine cancer vaccine specificity [5]. The major advantage of such approach is that the immune responses can be specifically directed toward minimal tumor antigens thus limiting autoimmunity and other side effects. On the contrary, major limitations of such an approach are represented by the need of MHC prediction binding algorithms for the selection of immunogenic peptides, the heterogeneity of MHC restriction, the down regulation of MHC class I molecules in many tumor types and, most importantly, the limited immunogenicity of peptides. The consequence of the latter aspect is that an optimal combination of tumor-specific associated antigens, adjuvant formulations and delivery system is crucial for the effective induction of anti-tumor immune response. 

In this framework, nanotechnology provides an effective tool for developing optimal antigen delivery strategies to improve the targeting of APCs and the efficacy of peptide-based cancer vaccines. Many types of nanoparticles have been developed by tuning specific parameters, such as particle size, surface properties (e.g., charge, hydrophilic property), geometry and kinetics [6,7]. In this review we report current strategies on the use of nanoparticles and nanomaterials in peptide-based cancer vaccine development.

## 2. Nanomaterials and Nanoparticles for Cancer Vaccines

Delivery systems in peptide-based vaccines should protect the peptides from degradation and actively or passively deliver them into APCs, aiming at inducing their maturation by interacting with elements of the innate immune system such as Toll-like receptors [8]. 

Nanoparticles have most of such properties, including prolonged biological activity, enhanced bioavailability, antigen protection from degradation and controlled antigen release. Different nanoparticles have specific chemical and physical properties affecting the interaction with target cells and include polymeric materials, liposomes, micelles, mesoporous silica nanoparticles (MSNs), gold nanoparticles (AuNPs) and virus nanoparticles. In addition, the delivery and the intra-cellular trafficking of nanoparticles can be optimized by selected modification of their surface (e.g., charge, structure, dimension and hydrophobicity) [9,10] and by incorporating cell-penetrating peptides, APC-specific cellular epitopes or immune-stimulant lipid moieties [11]. 

### 2.1. Liposome

**Figure 1:**
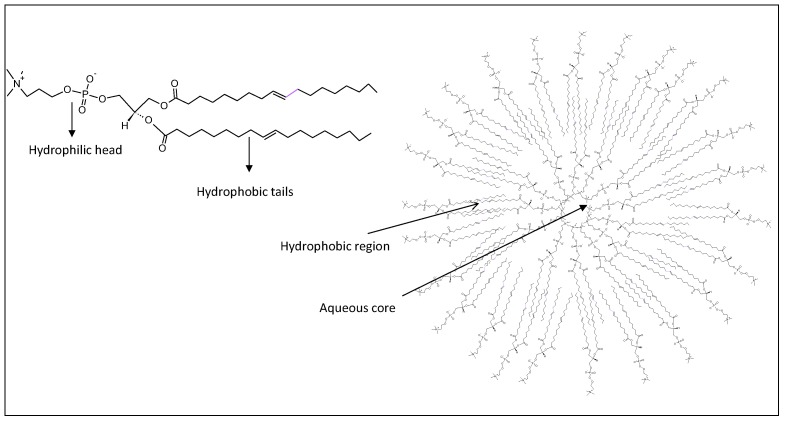
Schematic representation of liposome (right). Chemical structure of a generic phospholipid used to prepare the liposome (left).

Liposomes are spherical nanovesicles consisting of single or multiple phospholipid bilayers which, for their dual hydrophilic or hydrophobic characteristics, can incorporate hydrophilic antigens into the aqueous inner space or lipophilic components into the lipid bilayer (Figure 1) [12,13]. 

The liposome properties depend on their lipid composition, surface charge and size. Combination of different lipid types, or chemical modifications of the surface charge with cationic or anionic moieties, such as amine or carboxylic groups, can significantly enhance the immune response to carried cancer vaccines. Depending on the preparation process, liposomes have sizes between 0.5 microns to 5 microns but they can also been downsized in postproduction [12]. Drug encapsulation can be carried out by passive or active loading methods. It has been shown that the passive method requires controlled temperature and is time-dependent [14]. Generally, this method gives low encapsulation efficiency mostly for hydrophobic compound. While active methods, such as pH gradient or solvent-assisted active loading technology, are used for different types of molecules and consist in the preformation of liposomes containing a transmembrane gradient [15]. Surface functionalization of liposomes is an alternative method to conjugate peptides to liposomes and enhances cellular uptake, prevents lysosomal degradation and improves the stability of nanoparticles [16].

Liposomes have been successfully used as delivery systems for siRNA, DNA and protein or peptide antigens. Positively charged liposomes have been shown to induce stronger immune responses even at low doses, compared to negatively charged liposomes, since they can be more efficiently taken up by APC-like macrophages and DCs [17]. 

Gao et al. compared the antigen cross-presentation proficiency of cationic liposomes, composed of 3β-[N-(N’,N’-dimethylaminoethane)-carbamoyl]cholesterol (DC-Chol) and 1,2-dioleoyl-3- trimethylammonium-propane (DOTAP) with tertiary amine groups, with negatively charged liposomes composed of EPC/Chol/DSPE-mPEG. DOTAP/DC-Chol cationic liposomes induced an increased lysosomal pH in DCs and a reduced antigen degradation, thus promoting cross-presentation and cross-priming of CD8^+^ T-cell responses [18]. The study also evaluated the activity of nanoparticles at different concentrations. It has been shown that at low concentration DOTAP-CLs (20 μg/mL) promote cross-presentation but not at 100 or 500 μg/mL. On the contrary, DC-Chol-CLs promote cross-presentation at 100 μg/mL, but not at 500 μg/mL. Moreover, the high concentrations cause cytotoxicity that is one of the most limitations of using cationic liposomes.

Heuts et al. developed three liposome formulations to encapsulate 15 different long synthetic peptides (LSPs). The peptide containing the OVA SIINFEKL epitope was encapsulated in DOTAP:DOPC (dioleoyl-3-trimethylammonium propane:dioleyl phosphatidylcholine) liposome using three solvents and all three formulations were shown to activate CD8^+^ T-cells and antigen presentation by dendritic cells, suggesting the potential of such liposomes as delivery system for personalized cancer vaccines [19] (Table 1).

The combination of five lipids, including DMPC, DMPG, cholesterol, DOPE and MPLA, has been used to generate a liposomal carrier for peptide P5 (Lip-DOPE-P5-MPL) to be employed as effective cancer vaccine against breast cancer. To improve the P5 peptide encapsulation efficacy, liposomes have been chemically modified by conjugation with maleimide-PEG2000-DSPE and the formulation Lip-DOPE-P5-MPL was shown to elicit robust CTL response *in vitro* by efficient release of P5 peptide into APCs cytosol [14]. 

A peptide-CpG-DNA-liposome complex vaccine has been developed by Park et al. to treat pancreatic cancer. The study has been carried out in a mouse allograft model with TM4SF5 expressing pancreatic cells. Liposome complexes consisting of cyclic peptide and CpG-DNA co-encapsulated with DOPE:CHEMS (dioleoylphosphatidylethanolamine:cholesteryl hemi- succinate) induced the production of anti-hTM4SF5 antibodies and suppressed the growth of TM4SF5-expressing pancreatic cancer.

The same TM4SF5 cyclic peptide complexed with DOPE:CHEMS liposomes and CpG-DNA adjuvant was previously studied in metastatic HCC and colon cancer. Furthermore, the same liposome complexes showed a growth inhibition of colon tumors in a mouse lung metastasis model [20].

However, liposomes have some disadvantages, such as low encapsulation efficiency, limited solubility, propensity to phospholipid degradation, compound leakage and fusion. Nevertheless, this can be overcome modifying their properties by incorporating specific components such as cell-penetrating peptides (RRRRRRRR) or KALA peptides (WEAKLAKALAKALAKHLAKA LAKALKA) [37] or by surface functionalization [16]. Miura et al. demonstrated that the conjugation of the α-helical peptide KALA to OVA liposomes induced a much more potent OVA-specific MHC class I restricted antigen presentation in comparison with R8-OVA liposomes, which were previously reported to be efficient antigen carriers [21]. Such an improved efficacy seems to correlate with a more pronounced membrane-fusogenic activity of the α-helix structure in the KALA-OVA liposomes [37].

Razazan et al. produced DOPE-based pH-sensitive liposomes (DMPC-DMPG-Chol-DOPE) linking on their surface the monophosphoryl lipid A (MPL) adjuvant together with the breast cancer Gp2 peptide, derived from HER2 protein transmembrane domain (DMPC-DMPG- DOPE-MPL-Gp2). The DMPC-DMPG-DOPE-MPL-Gp2 vaccine was shown to enhance the therapeutic efficacy with an increased INF-γ production by splenocytes compared to the GP2 free peptide in a mice xenograft model. Importantly, the presence of MPL in the vaccine complex induces a Th1 immune response and activation of Toll-like receptor 4 (TLR4). In parallel, DOPE lipids in the cells undergo acidification (pH < 6.5) and destabilization with lamellar-to-hexagonal transition and release of carried molecules. The results of the study suggested that this formulation could be a potential vaccine candidate for HER2/neu breast cancers [38]. DMPC-DMPG-Chol-DOPE formulation has also been used in combination with MPL to conjugate the HER2 epitope, P435 (Lip + DOPE + MPL + P435). The conjugation of peptide on the surface of liposome was carried out through the binding to maleimide-Peg2000-DSPE via thioether binding. The size of obtained nanoparticles was between 159 and 183 nm, optimal for the delivery to lymph nodes. Lip + DOPE + MPL + P435 was shown to inhibit the most tumor growth in the TUBO mice model and extend the survival time [39]. 

In a recent study, Lay et al. reported that the combination with mannose and CpG-ODN, increases the efficacy of liposomes in stimulating DCs activation. The vaccination strategy was based on a liposomal vaccine (M/CpG-ODN-TRP2-Lipo), obtained by the assembling of the DC-targeting mannose and immune adjuvant CpG-ODN on the surface of liposomes loaded with melanoma-specific TRP2_180-188_ peptide. Such a combination showed an enhanced anti-melanoma effects in mice compared with a whole tumor cell lysate-based vaccine [40].

### 2.2. Polymeric Nanoparticles

**Figure 2:**
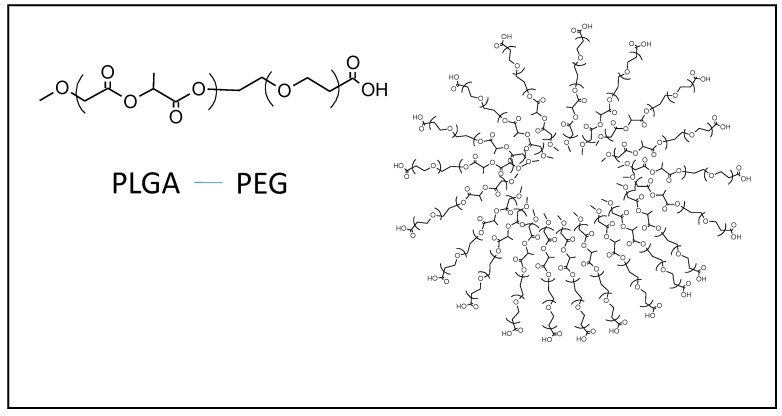
Schematic representation of polymeric nanoparticle (right). Chemical structure of polymer based on the combination of PEG and PLGA (left) used for the formation of the nanoparticle.

Polymeric nanoparticles have been developed as antigen delivery system and, in particular, poly(_D-L_-lactic-co-glycolide) nanoparticles (PLGA-NPs) are the best known model used in cancer vaccine together with PEG (Figure 2) [22,23]. 

Polymeric NPs show biological properties partially shared with liposomes, including high stability in biological fluids, tissue biocompatibility and formulation versatility. Several techniques have been used to produce such nanoparticles, and the final characteristics, size and structural organization, depend on the method and solvents chosen. The most common strategy to produce polymeric NPs is single or double emulsion-solvent evaporation. To incorporate hydrophobic molecules polymers and drugs are dissolved in an organic solvent, and then emulsifying this in water. Subsequently the obtained oil-in-water emulsion is lyophilized. While, hydrophilic drugs are first dissolved in water and then emulsified in organic polymer solution. The sizes are in the micron range but they can be reduced, as well as for liposome, by filtration methods [22].

Moreover, physicochemical properties (e.g., hydrophobicity, surface charge), drug release profile and biological behavior (e.g., bioadhesion, targeted drug delivery, improved cellular uptake) can be modulated by application of different polymeric materials and targeting ligands [24]. 

Besides PLGA, PEG and their combination, different polymers have been used in nanocarrier systems, including polycaprolactone, chitosan and dextran, cellulose, nanocrystal [41]. 

The efficacy of PLGA NPs and PLGA/PEI NPs (polyethylenimine) as peptide delivery system for therapeutic cancer vaccine development was assessed in an *ex vivo* cell culture model [23]. Flow cytometry as well as confocal laser scanning microscopy (CLSM) showed that PLGA/PEI NPs are more readily taken up by both human CD14^+^ monocytes and mouse Hepa 1-6 hepatoma cell line compared to PLGA NPs. Both NPs showed a clathrin-dependent as well as a caveolin-dependent internalization pathway and, once in the cells, they formed multivesicular endosomes (MVE). An *ex vivo* priming experiment showed that PLGA NPs are more efficient in delivering a non-self antigen (i.e., ovalbumin − OVA) to immature dendritic cells (imDCs), which fully matured inducing autologous naïve CD4^+^ T cells to differentiate to memory (i.e., central memory and effector memory) cells. Such a differentiation was associated with a Th1 phenotype. The same OVA antigen in a soluble form was unable to induce maturation of DCs, indicating that both NP formulations provided an intrinsic adjuvant effect combined to efficient antigen delivery [23].

Galiverti et al. have shown that conjugation of a HPV E7 synthetic long peptide to ultra-small polymeric nanoparticles (NPs) enhances the antitumor efficacy of therapeutic vaccination in different mouse models of HPV+ cancers. In particular, they demonstrated that the NP-E7LP vaccine formulation is capable, in comparison to the non-NP-conjugated free-E7LP, of eliciting a systemic immune response characterized by a larger pool of E7-specific CD8^+^ T cells producing activation-associated cytokines and granzyme B (GZB) [25].

In a recent study, PLGA nanoparticles have been used for the delivery of a immunogenic heteroclitic peptide (BCMA72-80) (Table 1). The heteroclitic BCMA72−80 [YLMFLLRKI] peptide is encapsulated in PLGA to improve antigen delivery and presentation, thereby inducing more robust polyfunctional BCMA-specific anti-tumor CD8^+^ CTL responses compared to vaccination with peptide alone [42].

It has been shown that polystyrene nanoparticles (PSNPs) are biocompatible, do not induce inflammation and induce CD8^+^T cell responses specific for the delivered peptide. Xiang et al., developed a nanoparticles based hSp17 peptide vaccine for the treatment of ovarian cancer. The immunogenicity of the formulation made with six hSp17 peptides conjugated to PSNPs has been compared with the hSp17 peptides adjuvanted in CpG. Both formulations induced similar levels of IgG in HLA-A2.1 transgenic mice, which were also comparable to the CpG adjuvanted formulation in C57BL/6 mice. Nevertheless, the PSNPs adjuvanted formulation in C57BL/6 mice induced much lower antibody response [43]. More recently, the same authors tested the immunogenicity of 24 peptides associated with most frequent gynecological malignancies (e.g., HPVE7, Survivin (SV) and Wilms tumor antigen 1 (WT1) conjugated with PSNPs). The results showed that the PSNPs-conjugated peptides were able to elicit a strong CD8^+^ T cell immune response, even when the same peptides adjuvanted with CpG failed. Therefore, PSNPs may represent an alternative vaccination strategy when conventional adjuvants are unable to elicit the desired CD8^+^ T cell reactivity [26].

### 2.3. Hydrogels, Nanogels, Micelles, Dendrimers

**Figure 3:**
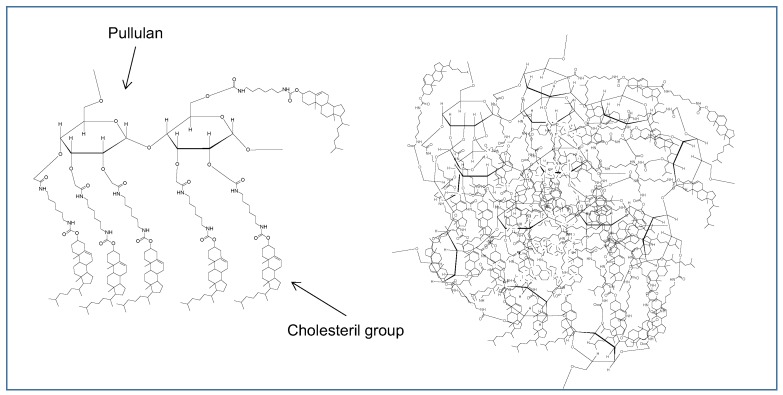
Schematic representation of cholesteryl pullulan polysaccharide (CHP)-nanogel (right). Chemical structure of (CHP) (left).

**Figure 4:**
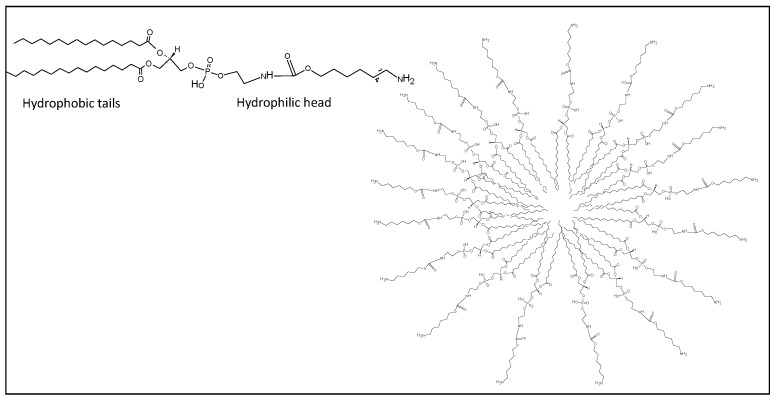
Schematic representation of micelle (right). Chemical structure of PEG-PE (left).

**Figure 5:**
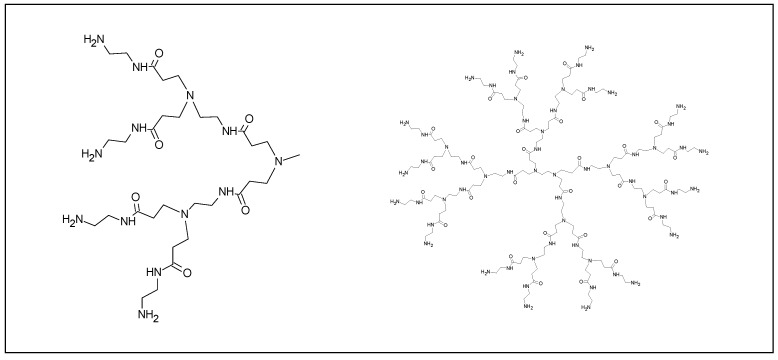
Schematic representation of dendrimer (right). Chemical structure of the monomer (left).

Another type of polymeric nanoparticles such as nanogels, dendrimers, hydrogels and micelles have been designed and used as vaccine delivery carriers, after conjugation with immune stimulants (Figure 3, Figure 4 and Figure 5). 

#### 2.3.1. Hydrogel Nanoparticles

Hydrogels are three-dimensional cross-linked polymer networks that absorb a large amount of water when placed in aqueous solution and their surface is appropriate for multivalent conjugation [27]. Hydrogels can be classified in three categories depending on their size: macroscopic hydrogels (size about millimeters), nanogels (sizes about nanometers) and microgels (size about micrometers). Different synthetic methods have been developed for the production of hydrogels, in particular cross-linking methods [28]. The properties of hydrogels can be chemically modified to regulate the solubility by functionalization with hydrophilic and hydrophobic polymers or other materials such as magnetic particles [44].

Other important modifications in the hydrogel delivery system were reported previously by Muraoka et al., encapsulating a synthetic long peptide antigen, derived from the human tumor antigen MAGE-A4, within a cholesteryl pullulan (CHP) nanogel. Indeed, it was demonstrated that the pullulan polysaccharide modified with cholesteryl group is able to form nanogels in the aqueous environment. In the same study was also shown the induction of strong anti-tumor immune response and CT26/MAGE-A4 tumor growth control when the MAGE-A4/CHP delivery system was adjuvanted CpG [45]. Similar results were obtained in the B16-F1 tumor model with nanogels delivering the KVPRNQDWL peptide derived from melanoma antigen gp100.

#### 2.3.2. Nanogel Nanoparticles

Polymeric nanogels are nanoparticles with hydrogels-like particles, with particle size of 100–200 nm. Like hydrogels, they can be chemically modified for improved efficacy. Cationic nanogels, with high uptake by DCs, have been developed using dextran, SLPs, containing CTL and CD4^+^ T-helper (help) epitopes, and polyinosinic-polycytidylic acid poly(I:C). The peptides, modified with a cysteine at N-terminus, were covalently conjugated via disulfide bonds to the polymeric network of cationic dextran nanogels. The *in vivo* study showed that the covalently conjugated peptide nanogels stimulate strong functional CD8^+^ and CD4^+^ responses in comparison to naked SLP and non-conjugated formulations, indicating the key role of reducible covalent bond for intracellular delivery of vaccine peptides [46]. Despite the existence of several studies reporting on hydrogel production, there are still many problems regarding the translation process, including the stability during the storage, the regulatory complexity and the cost [47].

#### 2.3.3. Polimeric Micelles

Polymeric hybrid micelles made of amphiphilic di-block copolymers, like PEG-phosphorethanolamine (PEG-PE) and polyethylenimine-stearic acid (PSA) conjugate, were reported to enhance the immunological potency of cancer vaccine models. The most common way to prepare micelles is oil in water emulsion or solvent evaporation. Generally, depending on composition, the sizes are between 2 to 20 nm. One of the principal differences with other nanoparticles, in particular with liposomes, is the stability of the resulting formulations. The critical micelle concentration (CMC), defined as the concentration of surfactants above which the micelles are spontaneous formed, increases in the presence of salts with consequently breakdown the micelles’ structures [48]. Moreover, in the blood stream micellar formulations are diluted in blood and tend to dissociate into monomers with immediate drug release. For these reasons several chemical modifications have been considered to improve the stability and the efficiency of micelles [49] and actually only a few are FDA approved [50].

The hybrid micelles were loaded with melanoma antigen peptide Trp2 (Table 1), cytosine-phosphate-guanine oligodeoxynucleotide (CpG ODN), and polyethylenimine-stearic acid (PSA) was added for positive charging in order to improve cellular uptake *in vitro*. Hybrid micelles HM50 showed more efficient targeting of popliteal draining lymph nodes (DLNs) and stronger induction of cytotoxic T-lymphocytes (CTLs) responses than free CpG and Trp2-PEG-PE micelles *in vivo* [51]. Polymeric micelles are currently considered to be a more exploratory nanoparticle carrier system for cancer immunotherapy. Indeed, these colloidal systems can selectively accumulate in solid tumors, showing improved loading capability, therapeutic efficacy and targeting ability by surface modification with tumor homing ligands and aptamers. Micelles have been developed using synthetic polymers as well as natural polysaccharides to deliver a variety of molecules, including, but not limited to drugs, proteins, peptides, DNA, SiRNA. In particular, micelles based on polysaccharides have shown promising results in cancer therapies [52].

The delivery of tyrosinase-related protein 2 peptide antigen and adjuvant to the lymph node in the B16F0 melanoma mouse model was performed using cationic diblock polymeric micelles. The resulting increased T lymphocyte anticancer activity indicates the efficiency of such micelles in treating cancer by improving the immune response [2].

#### 2.3.4. Dendrimer Nanoparticles

Dendrimers are hyper-branched polymers that possess versatile multivalent surfaces for interacting with surrounding surfaces [53]. In a recent application, multivalent glycodendrimers, generated using branched polyamidoamine (PAMAM) dendrimers, have been considered as scaffold for melanoma-specific gp100 SLPs. Ligands for the DC-SIGN and langerin have been added on the scaffold to evaluate the targeting efficacy to human dermal DCs and Langerhans cells (LCs). The strategy resulted in enhanced gp100 internalization and antigen-specific CD8^+^ T cell activation [29].

### 2.4. Inorganic Nanoparticles

**Figure 6:**
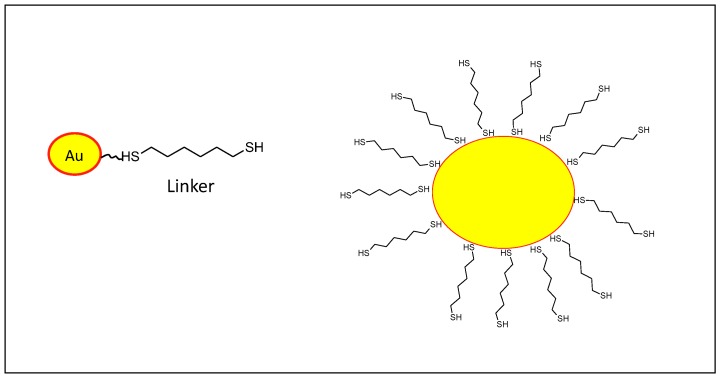
Schematic representation of gold (Au) nanoparticle modified with likers on the surface for further functionalization (right). Au Core (yellow) conjugated with a dithioether linker (left).

Inorganic nanoparticles have been used in various applications, such as bioimaging, sensors, drug delivery and therapeutics, and cancer immunotherapy due to their unique optical, physical, chemical, electronic and magnetic properties. The size, shape and surface properties of inorganic nanoparticles can be easily manipulated during the synthesis. Several inorganic nanoparticles are in preclinical stage as vaccine delivery systems, showing significant biostability. Furthermore, inorganic nanoparticle trafficking and cargo release can be internally or externally induced by factors like temperature, pH, metabolites, magnetic fields and/or light. Gold, iron oxide, aluminum-based nanoparticles, upconversion nanoparticles and mesoporous silica are all viable delivery systems for cancer vaccines (Figure 6).

Inorganic nanomaterials can form cores and provide scaffolding with unique structural and dynamic properties for other biomaterials, such as polymers and lipids, to construct robust and effective delivery vectors.

#### 2.4.1. Gold Nanoparticles

Gold nanoparticles (AuNPs) are biocompatible, not immunogenic and manufactured in diverse sizes and shapes [30]. AuNPs can be easily prepared from gold salt (H[AuCl4]) in water and their particle sizes are controllable (ranging from 1-100 nm diameter) [54,55].

AuNPs were functionalized with mucin-1 (MUC-1) glycoprotein and the resulting nano-constructions have been shown to induce the production of cytokines such as TNF-alpha, IL-6, IL-10, and IL-12 by peritoneal macrophages isolated from mice [31,32]. Glycosylated gold nanoparticles are constructed as synthetic cancer vaccines by conjugating them with tumor-associated (Tn) antigen glycans to induce strong antibody response specific for aberrant mucin glycans. The AuNPs modified with PEG25Tn25 and PEG80Tn2 showed the strongest immune stimulatory antibodies among all PEG-modified formulations *in vivo* [33]. To improve the therapeutic effect, AuNPs have been modified and combined with peptides. Fytianos et al. combined AuNPs with PEG, polyvinyl alcohol (PVA) or a mixture of both with either positive or negative surface charge. The results suggested that surface modification influenced uptake, with a high degree of internalization of (PEG+PVA)-NH2 and PVA-NH2 AuNPS [56]. It has been shown that the combination of AuNps with OVA peptide and the CpG adjuvant can improve the delivery of peptide and enhance the therapeutic effect in a B16-OVA tumor model [57].

Liang et al. reported a new nanoplatform of liposome-coated AuNPs modified with DCs specific aCD11c antibody for targeted delivery of adjuvant MPLA and melanoma antigen peptide TRP2180-188 (SVYDFFVWL). The nanoparticle system was shown to efficiently stimulate DCs, resulting in the maturation of DCs and activation of anti-tumor CD8^+^ T lymphocytes with tumor growth inhibition in both B16-F10 prophylactic and lung metastasis models [58].

#### 2.4.2. Iron Oxide Nanoparticles

Iron oxide nanoparticles can also be used as potent carriers for vaccine delivery and can be used as antitumor agents for cancer therapy. A very recent study described a superparamagnetic Fe_3_O_4_ as a delivery system of OVA, showing superior induction of both immune cell activation and cytokine production with effective control of tumor growth [59]. Indeed, magnetic nanoparticles can be directly controlled by an external magnetic field to significantly improve lymph node as well as intra-tumor retention [60].

#### 2.4.3. Aluminum Salts (Alum)

Alum is one of the few licensed adjuvants for human use and is included in several licensed vaccine formulations. It can form a depot at the injection site, inducing sustained release of antigen, and prolonging the interaction between antigen and immune cells, resulting in the induction of more potent antigen-specific B or T cell response [61].

Alum has been used as adjuvant in combination with CpG oligodeoxynucleotide (CpG) and innate defense regulator peptide HH2 for improving anti-tumor immune responses. The CpG-HH2 complex significantly enhanced the production of IFN-γ, TNF-α and IL-1β, promoted the uptake of antigen and strengthened the activation of p38, Erk1/2 and NF-κB *in vitro*, compared to CpG or HH2 alone [34]. Aluminum hydroxide nanoparticles (~100 nm) containing OVA was shown to have a strong *in vitro* immune activity and to delay tumor progression *in vivo* after immunization with a dose lower than traditional aluminum hydroxide (~9 μm) [35]. 

#### 2.4.4. Other Inorganic Nanoparticles

Multishell calcium phosphate (CaP) nanoparticles are considered ideal carriers for biomolecules as they can transport many molecules across the cell membrane and protect the encapsulated biomolecules against enzymatic degradation. In combination with CpG and viral antigens, CaP nanoparticles facilitate a strong activation of DCs and generation of virus-specific T cells. Application of functionalized CaP nanoparticles during chronic viral infection was sufficient to overcome barriers of T-cell exhaustion and supported the reinforcement of cytotoxic CD8^+^ T cells in contrast to the administration of soluble CpG and peptides. Heβe et al. demonstrated that the administration of HA-peptide and CpG functionalized CaP nanoparticles in murine xenograft tumor model expressing the viral antigen hemagglutinin (HA) was highly sufficient to enhance the antitumor T-cell response and to repress progression of tumor induced by HA-transduced CT26 cell line. In the same study, particles were loaded with a whole peptide pool derived from a primary tumor cell lysate, as a universal approach for individual cancer therapy, and a significantly decreased tumor growth was observed in immunized animals [62].

Fluorescent magnetic nanoparticles (α-AP-fmNPs) were constructed from iron oxide nanoparticles, indocyanine green and fusion peptides (α-AP). External stimulation by a magnetic pulling force significantly enhanced the migration of α-AP-fmNP-loaded DCs than control DCs both *in vitro* and *in vivo*. BMDCs treated with α-APOVA-fmNP showed enhanced *in vitro* CD8^+^ T-cell proliferation and cytokine IFN-γ production, as well as *in vivo* CTL response compared to nonmagnetic nanoparticle treated and non-treated controls [36]. 

Mesoporous silicas are solid materials featured by mesoporous structure encapsulation of biomolecules. Mesoporous silicas have been intensively studied as drug delivery systems due to the advantage of high surface area, tunable pore size and stable chemical/thermal properties [36,63]. MSNs can also be used as antigen carriers and/or adjuvants for cancer immunotherapy. 

Dellacherie et al. reported a vaccine strategy based on dendritic cell-recruiting Mesoporous Silica Rod (MSR) scaffolds to enhance T-cell responses against subunit peptide antigen. The small peptide OVA-derived SIINFEKL, modified with a cysteine at N-terminus, was covalently conjugated via thioether linkage to the MSRs. The conjugation enhanced the stability in subcutaneous tissue *in vivo*, and their ability to stimulate the OT-II T-cell, although a decreased antigen presentation by bone marrow derived dendritic cells was observed [64].

Cha et al., synthesized mesoporous silica nanoparticles with extra-large pores and tunable pore structure and particle size, which resulted in a high loading capacity of large biomolecules. Amine-modified XL-MSNs showed significantly higher loading of OVA and CpG. The XL-MSNs successfully delivered the antigen protein and TLR9 agonist into the cytosol and led to enhanced maturation and antigen presentation of DCs. Subcutaneously injected XL-MSNs were transported from the injection site to LNs in the animal study, with strong stimulation of antigen-specific cytotoxic T cells and tumor growth suppression in a prophylactic tumor model [65].

## 3. Virus-Like Particles, Proteinlike Nanoparticles and Peptides

### 3.1. Virus-Like Particles

**Figure 7:**
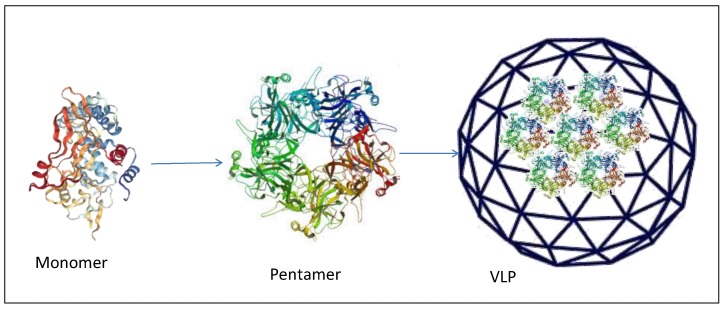
Schematic representation of VLPs (right). Modified from https://doi.org/10.1172/JCI85446.

Virus-like particles (VLPs) (Figure 7) have attracted significant interest as a cancer vaccine platform for inducing antigen-specific immune responses. Viral capsid proteins are able to self-assemble into particulate structures closely resembling the natural virus, from which they are derived. VLPs are replication as well as infection incompetent and can efficiently deliver antigens to APCs which are cross-presented in association with both MHC class I and class II eliciting both humoral and cellular immune responses [66]. 

VLPs are commercially available as preventive vaccines, such as the HPV cancer vaccines Cervarix^™^ (GSK) and Gardasil^®^ (Merck), inducing high levels of long lasting antibodies against HPV16 and HPV18 [67,68].

VLPs can be used as scaffolds and delivery systems for peptide vaccines. Storni et al. reported a VLPs vaccine that can eradicate established solid fibrosarcoma tumors in mice. CpG and the p33 peptide derived from the glycoprotein of lymphocytic choriomeningitis virus (LCMV) were packed into VLPs derived from the hepatitits B core antigen or bacteriophage Qβ. They found that such a vaccine induced high levels of peptide-specific CD8^+^ T cells that can eradicate solid fibroblastoma tumors [69].

In a recent study, Shukla et al. evaluated the potency of Cowpea mosaic virus (CPMV) nanoparticle-based cancer vaccine in several mouse models of HER2+ tumors in conjugation with the antigenic CH401 peptide (CPMV-CH401) (Table 1) derived from the extracellular domain of HER2 receptor. CH401-specific anti-sera elicited in immunized animals were shown to generate a strong cytotoxic effect against HER2 expressing cancer cells using a MTT assay [70].

A VLP system made of pyruvate dehydrogenase E2 protein nanoparticle showed great potential as a vaccine delivery platform for cancer. E2 nanoparticles co-delivering melanoma-associated gp100 epitope and CpG (CpG-gp-E2) induced a significantly enhanced *in vitro* CTL activity and cytokine IFN-γ secretion as well as an increased induction of T cell response and tumor growth control *in vivo* [71].

Recently, a nano-vaccine has been generated by coupling the p33 epitope to cucumber-mosaic virus (CuMV) coat protein. The CuMVTT-p33 nano-sized vaccine was next formulated with the micron-sized microcrystalline tyrosine (MCT) adjuvant and the formed depot effect was studied using confocal microscopy and trafficking experiments. The efficacy of such nanoparticles was next assessed in an aggressive transplanted murine melanoma model showing that CuMVTT-VLPs can efficiently and rapidly drain into the lymphatic system due to their nano-size of ~ 30 nm. The addition of the micron-sized MCT adjuvant of ~ 5 μM resulted in a local depot for the nanoparticles and a longer exposure to the immune system, eliciting an enhanced specific T cell response in the stringent B16F10p33 murine melanoma model. The study showed that the micron-sized MCT adjuvant was as potent as B type CpGs and superior to the alum adjuvant [72].

### 3.2. Protein and Peptide Particles

A large number of peptide epitopes able to induce strong, long-lasting humoral and cellular immunity against specific tumor or viral antigens, have been identified and studied. Chemical modifications to improve the peptide stability and/or to preserve the tertiary structure have been shown to enhance their immunogenicity *in vivo*. Nevertheless, a native conformational structure is not needed to elicit a T cell immunity given that the linear epitope of 8-10 amino acids is sufficient to be loaded onto the MHC-I molecules and presented to the T cell receptor. 

Naked peptides correspond to unmodified amino acid sequences used mostly in DC vaccines, in which expanded DCs are loaded with target peptide antigens *in vitro*, prior to re-introduction to the patient. In this case, delivery of concentrated naked peptides is sufficient for an efficient uptake and loading of DCs [73].

Stapled peptides consist of stabilized short sequences of amino acids modified by inserting hydrocarbon bonds into their natural alpha-helical conformation through side chains of amino acids [74]. Such modified peptides have not used as cancer vaccine approach yet, the only experimental model has been developed for inducing neutralizing Abs against the HIV-1 [75].

Different methods have been developed to improve metabolic stability of peptide antigens, for example acetylation of N-terminus, or amidation of C-terminus, modification of the structure of peptides into dendrimers, cyclization or replacement of some amino acids with unnatural amino acids, introduction of D-amino acids [71]. Efforts to improve the potency and quality of peptide vaccines include introduction of amphiphilic peptides, peptide fusions to Toll-like receptor (TLR) agonists, addition of powerful inflammatory adjuvants, and combinations with other immune modulators [76].

Covalent conjugation of peptides to carrier proteins is a standard method to improve the immunogenicity of peptides. One of the most used immunogenic peptide carriers, the Keyhole limpet hemocyanin (KLH), has been used in several clinical trials for anti-MUC1 vaccines in breast, ovarian, and colorectal cancer, although a phase III clinical trial failed to generate significant responses in breast cancer patients [77]. It has been shown that the conjugation of survivin_53-67_ peptide with KLH (SVN53-67/M57-KLH) stimulate an antitumor immune response against murine glioma *in vivo* as well as human glioma cells *ex vivo* [78].

More recently, cell-penetrating peptides (CPPs) have been generated to create multifunction drug delivery systems able to target specific cell compartments employing many different cargoes, including nanoparticles, proteins, liposomes and nucleic acids [71,79,80,81].

Two CPPs, HR9 and Cady-2, have been used in combination with natural adjuvant for an efficient *in vitro* and *in vivo* delivery of a complex antigen made of the Heat-shock-protein 27 and HCV NS3 DNA [82]. The Cady-2 improves the uptake of protein antigens, due to the presence in its sequence of hydrophobic and positively charged amino acids with two WW and two WF tandems, five Arg groups, but no negative charges (˜40% and ˜25%, respectively). Histidine-rich R9 (HR9: CH5-R9-H5C) contains poly-histidine and nona-arginine sequences and two cysteine residues which has been shown to increase gene delivery *in vitro*. The efficiency of cell penetration and immunogenicity was assessed in BALB/c mice, showing that heterologous prime/boost (G10: Hsp27-NS3 DNA + HR9/ rHsp27-NS3 protein + Cady-2) induced high levels of IgG2a, IgG2b, IFN-γ and Granzyme B and low levels of IgG1 and IL-5 directed toward Th1 responses [82].

Six cell penetrating peptides for *in vitro* and *in vivo* delivery of HPV16 E7 DNA and protein have been studied, with different formulation, as an antigenic model for therapeutic vaccine. The results showed that the immunization with the E7 protein/P28CPP nanoparticles, in both homologous and heterologous prime/boost vaccinations, induce the highest levels of IFN-γ and Granzyme B with complete protective and therapeutic anti-tumor effects [83].

## 4. Nanoparticles in Clinical Trials

Some of the nanoparticle vaccines have been evaluated in clinical trials for different cancers (reviewed in [84,85,86]). Among all, liposomes have been used extensively due to their versatility, including Tecemotide™ liposomes [87,88,89], AS15™ lipids [90,91,92], DepoVax™ liposomes [93,94,95], Iscomatrix [96], Cholesteryl pullulan (CHP) nanogels [97], and virus-like nanoparticles [98,99].

Most of these studies have shown induction of immune response specific to the antigen delivered by the nanoparticles, although none of the trials demonstrated statistically significant survival benefit. Such a lack of correlation between a significant immune response and poor clinical efficacy has not been yet explained but is suggestive of an immune response characterized by either wrong type, low magnitude, or not lasting. Moreover, nanoparticles may suffer from poor biodistribution and immune response may be hampered or suppressed by immunosuppressive tumor microenvironment (TME). All these aspects need to be addressed in detail in order to improve the efficacy of nanoparticle-based cancer vaccines (reviewed in [84,85,86] Table 2).

## 5. Conclusions

The development of nanoparticles in cancer vaccines is constantly expanding. Various type of nanoparticles including polymeric nanoparticles, liposome, virus, inorganic nanoparticles, natural nanoparticles such as polysaccharides, cell penetrating peptides and proteins have attracted great interest in the vaccine research fields. The nanoparticles can be tuned through modulating surface properties, size, shape and composition to enhance the immune responses against cancer. 

Moreover, adjuvants loaded into nanocarriers via hydrophobic or electrostatic interactions can further increase immunogenicity of tumor antigens. Furthermore, numerous nanocarriers designed for the delivery of conventional chemotherapeutics can be potentially applied to initiate and enhance immunogenic cell death and may further provoke the immune system against tumor cells when co-administered with cancer immunotherapeutics (Table 3). 

Nevertheless, despite encouraging results obtained in pre-clinical settings compared to soluble peptide antigens, the few clinical trials evaluating the efficacy of therapeutic cancer vaccines based on nanoparticles have not resulted in significant clinical outcomes. Such unsatisfactory results need to be thoroughly analyzed in order to identify strategies for improvements.

Moreover, translation into clinics of nanoparticles faces several challenges, including biological aspects, large-scale manufacturing, biocompatibility and safety, intellectual property (IP), government regulations, and overall cost-effectiveness in comparison to current therapies. 

Therefore, although nanotechnology in medicine has the potential to have a major impact on human health, a number of issues still need to be addressed and solved before nanoparticles will be translated into a clinically applicable cancer vaccine. 

## Figures and Tables

**Table 1:** Examples of nanoparticles formulated with peptides and adjuvants as cancer vaccine strategies.

Nanomaterials	Peptide Sequence	Adjuvant	Tumor	REF
Liposome	SLP OVA24: (SIINFEKL)	poly(inosinic-polycytidylic acid) (poly(I:C)	N/A	[16]
Liposome	p5 (ELAAWCRWGFLLALLPPGIAG), p373 (KIFGSLAFL), p435(IRGRILHDGAYSLTLQGLGIH), p1209 (SPPHPSPAFSPAFDNLYYWDQ)	CpG ODN 1826 (5-TCCATGACGTTCCTGACGTT-3) MPL	Breast cancer	[17,18]
Liposome	GP2 (654–662: IISAVVGIL)	MPL	Breast cancer	[18]
Liposome	GP2 (Ac-CGGGIISAVVGIL)	MPL	Breast cancer	[18]
Liposome	TM4SF5 peptide (TAGAYLLNRTLWDRCEAPPRVVPWNVT), hTM4SF5EC2-C: (TACAYLLNRTLWDRCEAPPRVVPWNcT)	CpG-DNA or flagella	HCC and colon cancer Pancreatic cancer	[19,20]
Liposome	TRP-2180–188 (SVYDFFVWL)	CpG-ODN	Melanoma	[21]
Polymeric nanoparticles	HPV E7 SLP (GQAEPDRAHYNIVTFCCKCDSTLRLCVQSTHVDIR)	CpG	HPV cancer	[22,23]
Polymeric nanoparticles	BCMA72−80 (YLMFLLRKI)		Myeloma	[24]
Polystyrene	hSp17111-142 (KEKEEVAAVKIQAAFRGHIAREEAKKMKTNSL), HPV16-E782−94 and HPV16-E641−65 (LLMGTLGIVCPICKQQLLRREVYDFAFRDLCIVYRDGN), WT1126−134 (RMFPNAPYL), SV90−124 (KKQFEELTLGEFLKLDRERAKNKIAKETNNKKKEF), SV2−36 (GAPTLPPAWQPFLKDHRISTFKNWPFLEGCACTPE)	CpG	Gynecological cancer	[25]
Hydrogel	(KVPRNQDWL)	CpG	Melanoma	[26]
Hydrogel	CTL (DEWSGLEQLESIINFEKLAAAAAK), Help (DEWEISQAVHAAHAEINEAGRE)	Poly(I:C)	Different cancers	[27]
Micelles	Trp2180-188 (SVYDFFVWL)	CpG ODN	Melanoma	[28]
Inorganic Nanoparticles	TRP2180-188 (SVYDFFVWL)	MPL	Melanoma	[29]
Inorganic nanoparticles	OVA (ISQAVHAAHAEINEAGR)		Colon adenocarcinoma	[30]
Inorganic nanoparticles	HA110–120 (SVSSFERFERFEIFPKESS) HA512–520 (YQILAIYSTVASSLVLL)	CpG	Colorectal cancer	[31,32]
Inorganic nanoparticles	APgp100 peptide (gp10025-33) (KVPRNQDWL)		Lymphoma	[33]
VLPs	gp33 (KAVYNFATM)	CpG	Fibrosarcoma	[34]
Viral nanoparticles (CPMV)	human163-182 (YQDTILWKDIFHKNNQLALT) rat167-186 (YQDMVLWKDVFRKNNQLAPV)		Breast carcinomas	[35]
VLPs	P33 (KAVYNFATMGGCK)	MCT	Melanoma	[36]

CpG = cytosine-phosphate-guanine, MPL = monophosphoryl lipid A, MCT = Microcrystalline tyrosine, Poly(I:C) = Polyinosinic-polycytidylic acid.Reference List.

**Table 2:** Nanoparticles evaluated in clinical trials.

Nanoparticles	Payloads	Clinical Stages	Indications	Ref
Liposome (L-BLP25)	MUC-1, tecemotide monophosphoryl lipid A	Terminated after phase III	NSCLC	[87,88,89]
Liposome (AS15)	MAGE-A3, CpG 7909 monophosphoryl lipid A	Terminated after phase III	Melanoma, NSCLC	[90,91,92]
Liposome (ISCOMATRIX)	NY-ESO-1	Terminated after Phase II	Melanoma	[96]
Liposome (DPX)	HLA-A2, Survivin polynucleotide	Phase I/II	Ovarian cancer	[93,94,95]
Cholesteryl pullulan (CHP)	HER-2 protein	Phase I/II	Esophageal cancer	[97]
Virus-like particles (VLPs)	Melan-A/MART-1, CpG	Phase I/II	Melanoma	[98,99]

**Table 3:** Biological, chemical and manufacturing characteristics of nanoparticles.

Nanoparticles	Advantages	Disadvantages	Manufacturing Limitations
Liposomes	Wide size range Ag encapsulated or on surface Hydrophobic or hydrophilic Ag FDA approved/Non-toxic Biodegradable	Reproducibility issues Oxidative Degradation	High cost
Polymeric Nanoparticles	Ag encapsulated or on surface Biodegradable FDA approved/Non-toxic Prolonged release of antigen	Ag degradation Ag burst release	Low scale-up
Hydrogels, Nanogels	Ag encapsulated Cell targeting with adhesion ligands Biodegradable	Difficult to handle Mechanically weak	Reproducibility Difficult to sterilize
Micelles	Biocompatible Biodgradable Easy to prepare and chemical modify	Highly unstable Must be fresh Cannot be stored	Reproducibility costs
Dendrimers	Hydrophobic Ags Tunable chemical and physical properties Cell targeting with adhesion ligands	No hydrophilic Ags Toxicity	High cost
Inorganic Nanoparticles(e.g., Gold NPs)	Size control Low cytotoxicity	Non-biodegradable Coating required	Low scale-up High cost
Mesoporous Silicas	Uniform pore diameter High surface area Electrostatic immobilization Suitable for covalent immobilization	pH sensitivity	High cost
VLPs	Prevention of off-target effects Stability	Immunogenicity of capsid Low encapsidation efficiency	Low stability
CPPS	Selective targeting Intracellular delivery Low toxicity Bioaction High stability	Resistance to drug Aggregation Endosomal entrapment	No limitation
